# Very Early Rehabilitation After Treatment with Intravenous Thrombolysis for Mild Acute Ischemic Stroke

**DOI:** 10.3390/neurolint17040060

**Published:** 2025-04-18

**Authors:** Rahul R. Karamchandani, Liang Wang, Dale Strong, Alexis A. Mulvaney, Jonathan D. Clemente, Jeremy B. Rhoten

**Affiliations:** 1 Department of Neurology, Neurosciences Institute, Wake Forest University School of Medicine, Advocate Health, Charlotte, NC 28203, USA; 2 Clinical Quality Analytics, Advocate Health, Charlotte, NC 28203, USA; liang.wang@atriumhealth.org (L.W.); edwin.strong@atriumhealth.org (D.S.); 3 Department of Physical Therapy, Advocate Health, Charlotte, NC 28203, USA; alexis.williams@atriumhealth.org; 4 Charlotte Radiology, Neurosciences Institute, Advocate Health, Charlotte, NC 28203, USA; jonathan.clemente@charlotteradiology.com; 5 Neurosciences Institute, Advocate Health, Charlotte, NC 28203, USA; jeremy.rhoten@atriumhealth.org

**Keywords:** early mobility, rehabilitation, intravenous thrombolysis, mild acute ischemic stroke

## Abstract

Background/Objectives: The optimal timing of rehabilitation after acute ischemic stroke is unclear. We studied neurological outcomes and safety of early mobilization (EM) within 24 h for patients receiving intravenous thrombolysis. Methods: This was a retrospective analysis of patients treated at a single Comprehensive Stroke Center from 6/2020–10/2024 with EM versus usual care. Patients were eligible for EM if they were treated with intravenous thrombolysis and had post-treatment National Institutes of Health Stroke Scale scores ≤ 5, and later, ≤10. Ordinal regression was performed to determine factors associated with a 90-day functional outcome benefit in the full cohort. Propensity scores were calculated for matched sample pairs to determine any shift towards better outcomes with EM. Results: Groups of 165 and 73 patients were treated with EM and usual care, respectively. Treatment with EM was not associated with improved 90-day neurological outcome (odds ratio [OR] for higher mRS 0.746, *p* = 0.265). The groups also had comparable rates of symptomatic intracranial hemorrhage, length of stay, and discharge disposition. In the propensity score analysis of 73 matched pairs, EM was comparable to usual care with respect to 90-day functional outcome (OR for higher mRS 0.891, *p* = 0.7). Conclusions: Mobilization within 24 h resulted in comparable rates of 90-day neurological function, symptomatic intracranial hemorrhage, and hospital length of stay in patients with mild ischemic stroke treated with intravenous thrombolysis. Future trials may further investigate the safety and efficacy of EM in alternate and larger patient cohorts.

## 1. Introduction

Stroke is a leading cause of death and disability in the United States and worldwide [1]. Rehabilitation after stroke, meanwhile, is an important component of recovery and is recommended by national guidelines [2]. Specifically, guidelines recommend that patients who suffer a stroke and for whom rehabilitation is appropriate should have structured and collaborative therapy in the post-acute setting (Class 1, Level of Evidence A) [2]. Early physical rehabilitation following a stroke aims to maximize functional recovery during a period of enhanced neuroplasticity while also reducing the risk of complications associated with inactivity, such as deep vein thrombosis and joint contractures [2,3].

The optimal timing and intensity of stroke rehabilitation is of some debate. American Heart Association/American Stroke Association guidelines recommend against mobilization within 24 h with “high-dose” rehabilitation based on the results of the AVERT clinical trial, which showed reduced odds of independent neurological function at 90 days with early and intensive rehabilitation compared with mobilization after 24 h [2,4]. It should be noted that while the early rehabilitation and usual care groups in AVERT were mobilized at 18.5 and 22.4 h, respectively, the intervention group received significantly longer daily, out-of-bed therapy than the usual care group (31 min versus 10 min, respectively; *p* < 0.0001) [4]. A dose-response sub-analysis of the trial showed that early rehabilitation sessions of short duration and higher frequency may be optimal [5]. Rates of favorable functional outcome and 50 m ambulatory status improved with more frequent sessions, while increasing time out of bed worsened outcomes [5].

Meanwhile, the safety of mobilization within 24 h in the subset of patients treated with intravenous (IV) thrombolysis is supported by multiple trials [4,6,7], though there are mixed data on the ability of early mobility (EM) to improve functional outcomes [4,7]. Arnold SM et al. showed that initiation of physical and occupational therapy within 24 h of receiving IV alteplase was safe and feasible [6], while Anjos et al. confirmed the safety of EM after thrombolysis in a larger trial, without showing short- or long-term functional outcome benefits [7].

Given these conflicting findings, we report the results of an institutional study examining the relationship of EM versus usual care on safety and functional outcomes in mild acute ischemic stroke patients treated with IV thrombolysis.

## 2. Materials and Methods

### 2.1. Study Design and Outcomes

This study was a retrospective analysis of a single Comprehensive Stroke Center and included acute ischemic stroke patients presenting from June 2020 to October 2024. The institutional project was intended to evaluate the safety and clinical outcomes of IV thrombolysis-treated stroke patients who received EM. Patients were eligible for EM if they had a National Institutes of Health Stroke Scale (NIHSS) score ≤ 5 after treatment with IV thrombolysis, per local standard of care. The eligibility criteria were expanded to include thrombolysis patients with NIHSS scores ≤ 10 in March 2022. Patients with occlusion of an intracranial large artery were not eligible. As this was a retrospective analysis using de-identified data, informed consent was not required by the local Institutional Review Board (IRB00082694).

The primary outcome was 90-day neurological function, measured by the modified Rankin Scale (mRS) score, where higher scores indicate worse outcome (range: 0–6) [8]. Exploratory outcomes included symptomatic intracranial hemorrhage (ICH), defined as worsening of 4 or more points on the NIHSS score within 36 h compared to the NIHSS score at presentation; hospital length of stay; and discharge disposition (home, acute rehabilitation, skilled nursing facility, or other).

Patients who were eligible for EM and received the intervention were compared to eligible patients who received usual care. Treatment assignments were made at the discretion of the therapist; eligible patients received EM per the ability of the therapist to perform the intervention within 24 h. All others were treated as per usual care.

The initial therapy evaluation consisted of a comprehensive neurological assessment, focusing on strength, sensation, vision, and cognition. In the EM group, mobilization occurred between 6 and 24 h post thrombolysis. Evaluations incorporated functional objective outcome measures, with the Postural Assessment Scale for Stroke as the standard measure [9]. Functional mobility was tailored to the patient’s abilities, with the goal of achieving the highest level of mobility as determined by the John’s Hopkins Highest Level of Mobility Scale [10]. Precautions included continuous monitoring of vital signs, ensuring blood pressure remains within prescribed parameters, and the use of assistive devices as necessary to optimize mobility and reduce the risk of falls.

### 2.2. Statistical Analysis

Baseline characteristics, including age, sex, race, premorbid mRS, comorbidities (hypertension, hyperlipidemia, diabetes, coronary artery disease, atrial fibrillation, and smoking), initial NIHSS score, presenting glucose, and computed tomography (CT) Alberta Stroke Program Early CT score (ASPECTS), were compared between patients receiving EM versus standard of care. Continuous variables were presented as median with interquartile range while categorical variables were displayed as a total number with percentages as counts with column percentages. *T*-tests, Chi-square tests, and Fisher’s exact tests were performed as appropriate for baseline variables of interest. A *p*-value < 0.05 was considered statistically significant.

Patient characteristics were compared between the good (90-day mRS 0–1) and poor (90-day mRS 2–6) groups. Since the 90-day mRS is an ordinal dependent variable, ordinal logistic regression was performed to identify factors associated with the primary outcome for the full cohort, adjusting for age, sex, race, comorbidities (hypertension, hyperlipidemia, diabetes, coronary artery disease, atrial fibrillation, and smoking), initial NIHSS score, presenting glucose level, and diagnosis of stroke (yes/no).

We also conducted propensity score matching analysis to reduce selection bias and confounding. Each patient’s propensity score was estimated using logistic regression, with treatment as the outcome. The covariates included age, sex, race, comorbidities (hypertension, hyperlipidemia, diabetes, coronary artery disease, atrial fibrillation, and smoking), initial NIHSS score, presenting glucose level, and diagnosis of stroke (yes/no). We then obtained matched pairs in the EM and usual care groups using nearest-neighbor matching. Ordinal logistic regression was performed to compare outcomes in the matched samples, using the 90-day mRS as the ordinal outcome and treatment as the predictor variable. All analyses were performed using Stata version 18 (StataCorp, College Station, TX, USA).

## 3. Results

During the study period, 343 patients were treated with IV thrombolysis. Of these, 247 were eligible for EM, with 9 missing 90-day mRS scores. The total cohort consisted of 118 patients with NIHSS ≤ 5 from June 2020 to February 2022 and 120 patients with NIHSS ≤ 10 from March 2022 to October 2024. Of the 238 eligible patients with 90-day mRS scores available, 165 were treated with EM and 73 with usual care. Median (interquartile range [IQR]) age was 64 (53–75) years in the EM group and 65 (56–75) years in the usual care group (Table 1). Median (IQR) NIHSS scores were 5 (3–10) and 6 (4–10) in the EM and usual care groups, respectively (Table 1).

Table 1 shows baseline characteristics for the cohort stratified by treatment group. Significant differences between treatment groups were seen for hyperlipidemia (35.2% in EM versus 56.2% in usual care; *p* = 0.002), diabetes (17% in EM versus 28.8% in usual care; *p* = 0.038), and IV thrombolysis to mobility time (0.7 days in EM versus 1.3 days in usual care; *p* < 0.001).

Table 2 shows patient characteristics stratified by 90-day outcomes (mRS 0–1 versus 2–6). Subjects in the good outcome group were younger (62 [52–73] years versus 69 [56–79] years; *p* = 0.002), had lower premorbid mRS scores (0 [0–0] versus 0 [0–2]; *p* < 0.001), lower rates of pre-existing hyperlipidemia (35.2% versus 54.4%; *p* = 0.005), less frequent diabetes (13.8% versus 34.2%; *p* < 0.001), and lower presenting glucose (125.3 milligrams/deciliter [mg/dL] versus 155.3 mg/dL; *p* < 0.001) and NIHSS scores (5 [3–8] versus 7 [5–12]; *p* < 0.001).

Comparing the full cohort of patients who received EM versus usual care, factors associated with higher 90-day mRS scores included older age (odds ratio [OR] 1.025, 95% confidence interval [CI] 1.005–1.045; *p* = 0.013), history of diabetes (OR 2.135, 95% CI 1.049–4.345; *p* = 0.037), and higher presenting NIHSS (OR 1.055, 95% CI 1.007–1.105; *p* = 0.025) (Table 3). Treatment with EM was not associated with improved outcomes (OR for higher mRS 0.746, *p* = 0.265). Symptomatic intracranial hemorrhage rates, hospital length of stay, and discharge disposition were not significantly different between the treatment groups.

Using propensity scores for the 73 matched pair samples, treatment with EM was comparable to usual care with respect to 90-day functional outcome (OR for higher mRS 0.891, 95% confidence interval 0.495–1.603; *p* = 0.7).

## 4. Discussion

In this retrospective analysis from a Comprehensive Stroke Center, minor ischemic stroke patients treated with IV thrombolysis who received EM had comparable 90-day functional outcomes compared to those managed with usual care. Symptomatic ICH, hospital length of stay, and discharge disposition were also similar between the groups.

The safety of EM has been shown in multiple prior studies. Subjects in the very early mobilization group of the AVERT trial had similar safety profiles compared with the usual care group [4]. A small study conducted in the United States of 18 patients treated with IV thrombolysis and mobilized with physical therapy within 24 h showed that the intervention was safe and feasible [6], as did a Brazilian study of 104 thrombolysis patients [7]. Rehabilitation started before day 3 was also shown to be safe in a large study conducted in Japan of patients treated with IV thrombolysis [11].

However, there are mixed data on the efficacy of EM. In addition to the primary outcome of the AVERT randomized trial, which demonstrated worse 90-day outcomes in the early and intensive rehabilitation group, a subgroup analysis showed a trend towards better outcomes with usual care compared with EM in patients treated with IV thrombolysis [4]. Momosaki R et al. showed that IV thrombolysis patients treated with rehabilitation within 0–2 days had improved 90-day functional outcomes compared to those treated beginning day 3 or later [11]. A study of 110 patients conducted in China demonstrated better outcomes in patients treated with rehabilitation within 24–48 h compared to beginning 72–96 h in a broad population of stroke patients [12]. A systematic review including studies published through June 2020 concluded that rehabilitation should begin after 24 h of stroke onset considering hemodynamics and safety [13]. An additional review suggested an opportunity for more specificity in clinical practice guidelines with respect to the evidence pertaining to EM, as well as greater flexibility given the dynamic nature of the evidence on the topic [14].

Our findings add to the existing body of literature supporting the feasibility and safety of mobility within 24 h in patients treated with thrombolysis, of which there are limited data from the United States [6]. AVERT enrolled patients in Australia, New Zealand, Malaysia, Singapore, and the United Kingdom [4], while Anjos JM et al., who demonstrated the safety of EM within 24 h of IV thrombolysis, conducted their study in Brazil [7]. Moreover, while an outcome benefit was not demonstrated in our study, the absence of worse 90-day neurological function with EM, in contrast to the original AVERT cohort, is a notable finding. In combination with no clear safety concerns, EM allows therapists to perform a higher volume of assessments, which may be of particular importance in the future as the national prevalence of stroke in the United States is increasing [1]. Overall, our findings reinforce the need for additional study of EM, including in patients with higher presenting NIHSS scores, those with intracranial large vessel occlusions with or without endovascular thrombectomy treatment [15], subgroups of thrombectomy patients (non-large core and large core), and in patients followed beyond the 90-day time window. Active and passive rehabilitation techniques, the latter of which are especially important when mobility may be restricted early in the recovery phase as this can promote neuroplasticity [16], also require additional study. The AVERT DOSE clinical trial, a multi-arm, multi-stage study enrolling both mild and moderate severity ischemic stroke patients within 48 h of symptom onset, will address some of these outstanding questions [17]. Lastly, multiple factors impact the ability to deliver EM [18], and future trials may focus on a more comprehensive approach to better implement early therapy. These factors may include staffing considerations, education, appropriate supply of equipment, and standardization of the therapy regimen [18], all of which may vary in different healthcare settings and thus create site-specific challenges.

The specific therapy regimen implemented with EM is an active area of investigation and is crucial to its potential success or failure as a treatment. The pre-specified AVERT sub-analysis suggested that shorter, more frequent therapy sessions, independent of age and NIHSS, may improve 90-day functional outcomes [5]. AVERT DOSE, meanwhile, will enroll patients within 48 h of stroke onset and include four separate groups receiving varying duration, intensity, and frequency of rehabilitation [17]. The primary outcome is 90-day mRS score 0–2, while secondary outcomes include all serious adverse events up to 6 months, as well as 6-month walking speed, quality of life, and a cost effectiveness analysis. While the original AVERT cohort included patients with a median NIHSS around 7 [4], and the median NIHSS in the EM group of our study was 5, AVERT DOSE will include both mild (NIHSS 0–7) and moderate (NIHSS 8–16) patients. Thus, the trial will help define the appropriate dosing regimen of rehabilitation in a more broad population of stroke patients and provide insight into longer-term outcomes. While the 48 h mark post-stroke is one of the key inclusion criteria for the trial, it will be interesting to note the percentage of patients enrolled within 24 h, as was investigated in AVERT and in our study. Equally intriguing will be the proportion treated with IV thrombolysis, as a subgroup analysis of AVERT showed a trend towards better outcomes with usual care than EM in this population [4]. It should be noted that AVERT DOSE will not include patients with hemorrhage strokes, although a prespecified subgroup analysis of AVERT showed better outcomes with usual care than EM in patients presenting with an ICH.

Our study is limited by the retrospective design of the analysis, subjecting the findings to multiple potential biases, including selection, recall, misclassification, and attrition bias (nine missing 90-day mRS scores). One source of selection bias pertains to the treatment group assignments, which were based on the ability of the therapist to perform a rehabilitation assessment within 24 h rather than by a systematic method. The small sample size, though comparable to other studies with functional outcome as the primary endpoint [7,12], may have limited our ability to detect a difference in 90-day mRS scores. Only one symptomatic ICH was observed in our study, and additional adverse events, such as falls and hemodynamic instability, would also have been informative to report if these data were available. Additional confounders may have impacted our findings, such as rehabilitation adherence and caregiver support, though we controlled for available and relevant covariates in the regression models and created matched samples with propensity scoring to strengthen the analysis. Although definitions of minor stroke differ in the literature [19], our study population largely consisted of patients with lower presenting NIHSS scores who may be expected to have good outcomes irrespective of reperfusion treatment or EM, limiting the generalizability of our findings to a broader population. We did not study patients with large vessel occlusion strokes, though this group will warrant additional investigation, particularly given the expanding indications for endovascular thrombectomy [20]. Hemorrhagic stroke patients were also not included, though they are also a group of interest, particularly given the results of the AVERT sub-analysis suggesting better outcomes with usual care than EM. Not all patients treated with IV thrombolysis are ultimately diagnosed with a stroke, though this was the case in over 80% of our cohort, reflecting real-life practice at a Comprehensive Stroke Center. While the therapy regimen was standardized to an extent at our center, alternative protocols, such as shorter-duration and higher-intensity treatments described in the AVERT sub-analysis that produced better outcomes, may have yielded different results. Also, the absence of specific information on duration, intensity, and frequency of rehabilitation in our trial on a patient-level basis makes it challenging to directly compare to alternate regimens. Lastly, as our findings differ from national guidelines and support the implementation of EM within 24 h in thrombolysis patients without presenting large vessel occlusion, we are hopeful that future clinical practice guidelines will be able to quickly and easily integrate new information in an ever-dynamic field.

## 5. Conclusions

In conclusion, minor ischemic stroke patients treated with IV thrombolysis who received EM had comparable 90-day functional outcomes, symptomatic ICH rates, length of stay, and discharge disposition to those receiving usual care. Additional study is required to determine whether EM is safe and provides a functional outcome benefit in alternate and broader populations of ischemic and hemorrhagic stroke patients; the appropriate dose, duration, and frequency of rehabilitation post-stroke; and the most comprehensive strategy to better implement early rehabilitation.

## Figures and Tables

**Table 1:** Baseline patient characteristics.

	Early Mobility (*n* = 165)	Usual Care (*n* = 73)	*p*-Value ^a^
Age, years, median (IQR)	64.0 (53.0–75.0)	65.0 (56.0–75.0)	0.54
Sex, male, *n* (%)	88 (53.3%)	42 (57.5%)	0.55
Race, *n* (%)			0.67
Black	54 (32.7%)	21 (28.8%)	
White	105 (63.6%)	48 (65.8%)	
Other/unknown	6 (3.6%)	4 (5.5%)	
Premorbid mRS, median (IQR)	0 (0–0)	0 (0–0)	0.91
Hypertension, *n* (%)	101 (61.2%)	48 (65.8%)	0.50
Hyperlipidemia, *n* (%)	58 (35.2%)	41 (56.2%)	0.002
Diabetes mellitus, *n* (%)	28 (17.0%)	21 (28.8%)	0.038
Coronary artery disease, *n* (%)	18 (10.9%)	9 (12.3%)	0.75
Atrial Fibrillation, *n* (%)	14 (8.5%)	4 (5.5%)	0.60
Smoking, *n* (%)	68 (41.2%)	28 (38.4%)	0.68
Initial NIHSS, median (IQR)	5.0 (3.0–10.0)	6.0 (4.0–10.0)	0.41
Glucose (mg/dL), mean ± SD	130.8 (51.8)	145.4 (76.2)	0.087
CT ASPECTS, median (IQR)	10.0 (10.0–10.0)	10.0 (10.0–10.0)	0.65
LKW to IV thrombolysis (days), median (IQR)	0.1 (0.1–0.1)	0.1 (0.1–0.1)	0.97
Bolus to mobility (days), median (IQR)	0.7 (0.5–0.8)	1.3 (1.1–1.5)	<0.001
Hospital length of stay (days), mean ± SD	4.5 (4.3)	4.7 (3.6)	0.75
Symptomatic intracranial hemorrhage, *n* (%)	0 (0%)	1 (1.4%)	0.31
Discharge diagnosis of stroke, *n* (%)	139 (84.2%)	58 (79.5%)	0.37
Discharge disposition, *n* (%)			0.25
Home	142 (86.1%)	57 (78.1%)	
Acute rehabilitation	16 (9.7%)	11 (15.1%)	
Skilled nursing facility/LTAC	5 (3.0%)	2 (2.7%)	
Other	2 (1.2%)	3 (4.1%)	

^a^ *T*-test, Chi-square test, rank-sum test, and Fisher’s exact test were used to calculate the *p*-value. n, number; IQR, interquartile range; NIHSS, National Institutes of Health Stroke Scale; mg, milligrams; dL, deciliter; SD, standard deviation; CT, computed tomography; ASPECTS, Alberta Stroke Program Early Computed Tomography Score; LKW, last known well; IV, intravenous; LTAC, long-term acute care; mRS, modified Rankin Scale.

**Table 2:** Characteristics stratified by 90-day functional outcomes.

	Good Outcome (mRS 0–1) *n* = 159	Poor Outcome (mRS 2–6) *n* = 79	*p*-Value ^a^
Age, years, median (IQR)	62.0 (52.0–73.0)	69.0 (56.0–79.0)	0.002
Sex, male, *n* (%)	83 (52.2%)	47 (59.5%)	0.29
Race, *n* (%)			0.30
Black	54 (34.0%)	21 (26.6%)	
White	100 (62.9%)	53 (67.1%)	
Other/unknown	5 (3.1%)	5 (6.3%)	
Premorbid mRS, median (IQR)	0 (0–0)	0 (0–2)	<0.001
Hypertension, *n* (%)	96 (60.4%)	53 (67.1%)	0.31
Hyperlipidemia, *n* (%)	56 (35.2%)	43 (54.4%)	0.005
Diabetes mellitus, *n* (%)	22 (13.8%)	27 (34.2%)	<0.001
Coronary artery disease, *n* (%)	17 (10.7%)	10 (12.7%)	0.65
Atrial Fibrillation, *n* (%)	10 (6.3%)	8 (10.1%)	0.29
Smoking, *n* (%)	63 (39.6%)	33 (41.8%)	0.75
Initial NIHSS, median (IQR)	5.0 (3.0–8.0)	7.0 (5.0–12.0)	<0.001
Glucose (mg/dL), mean ± SD	125.3 (47.7)	155.3 (77.0)	<0.001
CT ASPECTS, median (IQR)	10.0 (10.0–10.0)	10.0 (10.0–10.0)	0.99
LKW to IV thrombolysis (days), median (IQR)	0.1 (0.1–0.1)	0.1 (0.1–0.1)	0.31
Bolus to mobility (days), median (IQR)	0.8 (0.6–1.0)	0.8 (0.7–1.1)	0.087
Symptomatic intracranial hemorrhage, *n* (%)	1 (0.6%)	0 (0%)	0.99
Discharge diagnosis of stroke, *n* (%)	127 (79.9%)	70 (88.6%)	0.09

^a^ *T*-test, Chi-square test, rank-sum test, and Fisher’s exact test were used to calculate the *p*-value. mRS, modified Rankin Scale; *n*, number; IQR, interquartile range; NIHSS, National Institutes of Health Stroke Scale; mg, milligrams; dL, deciliter; SD, standard deviation; CT, computed tomography; ASPECTS, Alberta Stroke Program Early Computed Tomography Score; LKW, last known well; IV, intravenous.

**Table 3:** Ordinal regression analysis for higher 90-day mRS in full cohort.

	Odds Ratio	95% CI	*p*-Value
Early Mobility	0.746	0.446–1.249	0.265
Age	1.025	1.005–1.045	0.013
Male sex	1.375	0.843–2.241	0.202
Race			
Caucasian	0.971	0.545–1.730	0.92
Other/Unknown	1.711	0.544–5.382	0.358
Hypertension	0.764	0.426–1.371	0.367
Hyperlipidemia	1.471	0.860–2.518	0.159
Diabetes	2.135	1.049–4.345	0.037
Coronary Artery Disease	0.633	0.278–1.441	0.276
Atrial Fibrillation	1.578	0.608–4.094	0.348
Smoking	1.261	0.769–2.068	0.359
Presenting NIHSS	1.055	1.007–1.105	0.025
Glucose	1.002	0.998–1.007	0.366
Final diagnosis of stroke	1.639	0.818–3.286	0.164

mRS, modified Rankin Scale; CI, confidence interval; NIHSS, National Institutes of Health Stroke Scale.

## Data Availability

The original contributions presented in this study are included in the article. Further inquiries can be directed to the corresponding author.

## Abbreviations

The following abbreviations are used in this manuscript:

IV	Intravenous
EM	Early Mobility
NIHSS	National Institutes of Health Stroke Scale
mRS	Modified Rankin Scale
ICH	Intracranial Hemorrhage
CT	Computed Tomography
ASPECTS	Alberta Stroke Program Early Computed Tomography Score
IQR	Interquartile range
mg	Milligrams
dL	Deciliter
OR	Odds ratio
CI	Confidence interval
